# Comprehensive Protocols for Detecting Xenotransplantation-Relevant Viruses

**DOI:** 10.3390/mps8050109

**Published:** 2025-09-12

**Authors:** Hina Jhelum, Benedikt B. Kaufer, Joachim Denner

**Affiliations:** Institute of Virology, Free University Berlin, 14163 Berlin, Germany; hina.jhelum@fu-berlin.de (H.J.); benedikt.kaufer@fu-berlin.de (B.B.K.)

**Keywords:** xenotransplantation, virus safety, porcine cytomegalovirus/porcine roseolovirus, porcine endogenous retrovirus, PCR, real-time PCR, Western blot assay

## Abstract

Xenotransplantation using pig cells, tissues, or organs is advancing toward clinical application to address the shortage of human donor organs for treating organ failure. However, this emerging technology carries the risk of transmitting pathogenic porcine microorganisms, particularly viruses. The recent transmission of a porcine herpesvirus to the first human recipient of a pig heart highlights the urgent need for more rigorous screening of donor pigs. To identify potentially pathogenic porcine viruses, highly sensitive and specific detection methods are required. PCR-based techniques able to detect porcine cytomegalovirus/porcine roseolovirus (PCMV/PRV), hepatitis E virus (HEV), porcine circoviruses (PCV1-4), porcine lymphotropic herpesviruses (PLHV-1-3), porcine endogenous retroviruses (PERVs), porcine parvovirus (PPV), Torque teno sus viruses (TTSuV1,2), atypical porcine pestivirus (APPV) and SARS-CoV-2 were established. Immunological assays that detect antibodies as indirect indicators of infection were established for PCMV/PRV, HEV, PLHVs and PERVs. Since most veterinary laboratories focus on detecting viruses that are pathogenic to pigs and cause economic losses to the swine industry, screening for viruses relevant to xenotransplantation should be conducted in specialized virological diagnostic units. In this context, we present a complete collection of the newest and detailed protocols for comprehensive viral screening, along with guidance on how to implement these methods effectively.

## 1. Introduction

Xenotransplantation using pig cells and organs has made significant progress in recent years. First, there has been a remarkable increase in the survival time of pig xenotransplants in preclinical trials involving transplantation into non-human primates [1,2,3]. Second, successful transplantations of pig kidneys and hearts into brain-dead human recipients have been achieved [4,5]. Third, pig islet cells, as well as pig kidneys and hearts, have been transplanted for the first time into living human patients [6,7,8,9,10]. The donor pigs are genetically modified to reduce hyperacute and cell- and antibody-mediated rejection of the xenotransplant. To prevent transmission of porcine viruses, more or less comprehensive screening procedures have been implemented. Despite this effort, porcine viruses have been transmitted to non-human primate recipients during preclinical trials; these transmitted porcine viruses include the porcine cytomegalovirus, which is actually a porcine roseolovirus (PCMV/PRV) [11,12,13], and the porcine circovirus 3 (PCV3) [14]. The transmission of PCMV/PRV has been associated with fatal consequences. In non-human primates, it significantly reduced the survival time of transplanted pig organs [11,12,13]. PCMV/PRV was also transmitted to the first human recipient of a pig heart and contributed to the patient’s death despite the fact that the virus does not directly infect non-human primate or human cells [7].

On the other hand, viruses such as porcine respiratory coronavirus (PRCV), porcine reproductive and respiratory syndrome virus (PRRSV), porcine parvovirus (PPV), swine encephalomyocarditis virus (EMCV) and equine encephalomyelitis viruses (Venezuelan, Western and Eastern; VEEV, WEEV and EEEV) were found present in pigs used for the transplantation of encapsulated islet cells into diabetic cynomolgus monkeys [15], and PHLV1/2 were detected in pigs used for pig heart transplantation into baboons [13], but no transmission of these viruses was observed in the trials.

To ensure the safety of xenotransplantation, it is essential to first eliminate all viruses pathogenic to pigs, such as PRRSV, foot-and-mouth disease virus (FMDV), porcine parvovirus (PPV), porcine epidemic diarrhea virus (PEDV) and swine vesicular disease virus (SVDV), among others. In addition, viruses known to infect both pigs and humans must also be targeted for elimination. These include influenza virus, Nipah virus, hepatitis E virus, and various arboviruses such as Eastern equine encephalitis virus (EEEV), Japanese encephalitis virus (JEV) and vesicular stomatitis virus (VSV). However, it is important to note that most of these viruses are not endemic in Europe and North America [16].

Particularly important are viruses known to cause zoonotic infections in humans, such as hepatitis E virus genotype 3 (HEV3) [17]. Most HEV3 infections in humans are asymptomatic, especially in healthy individuals. When symptoms do occur, they can include fever, fatigue, nausea and jaundice. Humans are typically infected through consumption of undercooked or raw pork products (e.g., liver, sausages) or through direct contact with infected animals or their waste. However, immunocompromised individuals (e.g., organ transplant recipients) may develop chronic HEV infection, potentially leading to progressive liver disease or cirrhosis. Patients with pre-existing liver conditions are at greater risk of severe outcomes (for review, see [17]). Different RT-PCRs for detecting HEV3 were generated and compared [18], and the most sensitive methods were applied later [19]. In addition, an immunological method, a Western blot assay using different recombinant viral proteins, was established and applied [18].

PCMV/PRV does not infect healthy humans under normal conditions. Hence, it is not zoonotic, but it can cause disease in the context of xenotransplantation. Therefore, it should be classified as a xenozoonotic virus. We recognized early on that this virus significantly reduced the survival time of non-human primates transplanted with PCMV/PRV-positive organs [13]. In 2016, we reported the transmission of PCMV/PRV to a baboon following heart transplantation, even though the virus had not been detected in the donor animal—an occurrence later mirrored in the first human heart transplant involving a virus-positive organ [20].

We developed PCR-based methods, including conventional PCR, nested PCR and, most notably, real-time PCR, which proved to be the most sensitive and specific method for virus screening [21,22]. In addition, we introduced an immunological method in 2016 capable of detecting latent infections [23]. We also determined the optimal time points for applying each detection method [22].

Regarding all other viruses, their role in xenotransplantation remains unclear. Porcine circovirus 1 (PCV1) is non-pathogenic in pigs but was nonetheless included in the analysis. In contrast, porcine circovirus 2 (PCV2) can cause serious diseases in pigs, known collectively as PCV2-associated diseases (PCVDs), and should therefore be eliminated [23]. Although PCV2 has been found as a contaminant in rotavirus vaccines, evidence suggests it does not infect immunocompetent humans and is not considered zoonotic (for review, see [24]). Porcine circovirus 3 (PCV3), on the other hand, is pathogenic and has been linked to porcine dermatitis and nephropathy syndrome (PDNS), reproductive failure and multisystemic inflammation [25]. Recently discovered human circoviruses [26] are closely related to PCV3, which is of particular concern.

Although PLHV-1 to -3 are widespread in pigs, no association with any pig diseases has been reported so far (for review, see [24]). PLHV-1 has been detected in genetically modified donor pigs used in preclinical xenotransplantation studies; however, unlike PCMV/PRV, it was not transmitted to the recipient [13]. Given that PLHVs are related to bovine and ovine gammaherpesviruses—which, like PLHVs, are apathogenic in their natural hosts but can cause severe disease when transmitted to other species (for review, see [27])—it is advisable to use PLHV-free donor pigs to ensure safety of xenotransplantation.

Porcine endogenous retroviruses (PERVs), which are integrated into the genome of all pigs, cannot be eliminated using conventional elimination strategies. However, several approaches have been developed to prevent PERV transmission, including vaccines, small interfering RNA (siRNA) and genome editing techniques that inactivate all proviral copies in the pig genome [28]. Notably, no PERV transmission has been observed in any preclinical trials involving non-human primates or in clinical trials—despite the fact that the above-mentioned prevention strategies were not employed [28]. Meanwhile, all proviruses in pig cells have been inactivated using CRISPR/Cas [29], and using this method, healthy pigs have been generated [30].

When testing recipients for PERV infection, it is essential to consider microchimerism—the presence of pig cells, which naturally carry PERV sequences in their genome, distributed throughout the recipient’s body [31]. To distinguish a true PERV infection from mere microchimerism, sophisticated PCR methods and immunological assays have been developed and validated [32].

Provided that pig-pathogenic viruses have been eliminated due to their detrimental effects on donor pig health, we selected the following viruses as xenotransplantation-relevant [33]: PCMV/PRV, HEV, porcine circoviruses (PCV1-4), porcine lymphotropic herpesviruses (PLHV-1-3) and porcine endogenous retroviruses (PERVs). In addition, we developed detection methods for porcine parvovirus (PPV) [33], Torque teno sus virus (TTSuV1,2) [33], atypical porcine pestivirus (APPV) [34] and SARS-CoV-2 [19]. It was subsequently shown that SARS-CoV-2 does not infect pigs; however, this may change [35].

It is important to emphasize that our comprehensive detection systems not only employ highly specific and sensitive methods, either PCR-based or immunological, but also rigorously address critical factors such as sample generation, sample preparation, sample origin, timing of sample collection and the inclusion of appropriate negative and positive controls [36]. Since most veterinary laboratories focus primarily on viruses that are pathogenic to pigs and cause economic losses in the pig industry, specialized virological diagnostic units are required to screen for xenotransplantation-relevant viruses [37]. These specialized screening methods have been previously described and validated using pigs bred for xenotransplantation and their recipients [13,14], German slaughterhouse pigs [19] and various minipig breeds [18,33]. Special attention has been paid to the development of methods for detecting PCMV/PRV [21,22,33], PERV [28,38] and HEV3 [18]. In particular, the detection of PERV-C, which should be eliminated to prevent the emergence of high-titer PERV-A/C recombinants, is difficult, but reliable methods were developed [38].

In addition to the real-time PCR methods for detecting PCMV/PRV described here, a highly sensitive nested PCR method was also developed, and it can be used in parallel [21]. As these methods have been continuously refined, we present here a comprehensive and up-to-date collection of all detection methods for all relevant viruses in detailed form, enabling their effective use in screening pigs designated for xenotransplantation.

## 2. Experimental Design

These protocols have been developed for sensitive detection of porcine viruses relevant for xenotransplantation in cell cultures, donor pigs and recipients.

### 2.1. Samples from the Animals

In general, blood, isolated PBMCs and tissue samples can be used for detecting viruses by PCR-based methods. In some cases, non-invasively taken samples such as oral swabs and anal swabs can also be used; the details of the collection of the samples and the isolation of DNA from anal and oral swabs were described earlier [39]. However, the limitations of using oral and anal swabs are well known [40]. Blood is the best available material to analyze; it should be collected in EDTA tubes to prevent coagulation. Isolation of PBMCs will concentrate the cellular test material. Upon arrival of the blood, it should be immediately processed for PBMC isolation, and the plasma can be stored at −20 °C. For some viruses, such as PCMV/PRV [41] and PERV [42], stimulation of the PBMCs with mitogens may enhance the detection of the viruses. The methods used to treat PBMCs with mitogens in order to increase virus expression and facilitate virus detection are described in [41,42]. However, in the case of latent herpesviruses such as PCMV/PRV and PLHV, the virus may not be detected in blood or tissue samples by PCR methods. In this case, immunological methods such as immunohistochemistry can be used to detect PCMV/PRV antigens [41] and should be used to detect antibodies in sera, which might indicate infection [22,23]. Sera from donor pigs and recipients can be used for detection of antibodies in Western blot assays and ELISAs. Tissues, cells and sera can be stored frozen until testing.

### 2.2. Materials

The main materials are listed here; further details of the materials required for the PCR-based and immunological assays will be described in the descriptions of the procedures.

1. Pancoll human medium (PAN-Biotech GmbH, Aidenbach, Germany).
2. DNA and RNA isolation kits (QIAGEN, Hilden, Germany):
  2.1 DNeasy Blood & Tissue kit;
  2.2 QIAamp DNA FFPE Tissue Kit;
  2.3 RNeasy DSP FFPE Kit;
  2.4 RNase-Free DNase Set;
  2.5 RNeasy Plus Mini kit.
3. Ethanol, methanol (both from Carl Roth, Karlsruhe, Germany).
4. DreamTaq DNA polymerase (Thermo Fisher Scientific, Waltham, MA, USA).
5. PVDF membrane (0.2 µm; Carl Roth, Karlsruhe, Germany).

### 2.3. Equipment

The main equipment required for the PCR-based and immunological assays is listed here; further details will be described in the descriptions of the procedures.

1. Microcentrifuge Heraeus FRESCO 21 (Thermo Fisher Scientific, Waltham, MA, USA);
2. NanoDrop Spectrophotometer (Peqlab Biotechnologie GmbH, Erlangen, Germany);
3. Biometra TRIO cycler (Analytik Jena, Jena, Germany);
4. qTOWER^3^ G qPCR cycler (Analytik Jena, Jena, Germany);
5. Gel electrophoresis chamber (Bio-Rad, Hercules, CA, USA);
6. Semi-dry electro blotter (Peqlab Biotechnologie GmbH, Erlangen, Germany);
7. Imaging device (Peqlab Biotechnologie GmbH).

## 3. Procedure

### 3.1. Nucleic Acid Isolation

#### 3.1.1. Isolation of PBMCs from Blood Samples

1. Place 5 mL of lymphocyte separation medium (Pancoll human medium, PAN-Biotech GmbH, Aidenbach, Germany) in a 15 mL reaction tube. Hold the tube at a 45° angle.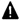 CRITICAL STEP: Slowly layer 5 mL of diluted blood (1:1 ratio of media to blood) on top using the pipetting aid, release very slowly and close the lid.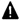 CRITICAL STEP: Centrifuge at 900× *g*, 25 min, 21 °C (switch off the brake).

Deceleration should be 0, as it is important for gradient formation.

4. The following layers are obtained after centrifugation (from bottom to top):
  - Erythrocytes;
  - PBMCs, white milky layer;
  - Plasma, yellow liquid.
5. If desired, transfer the plasma without disturbing the PBMC layer to a 1.5 mL reaction tube and store it at −20 °C.
6. Carefully aspirate the PBMC layer with a pipette and transfer it into a new 15 mL reaction tube.
7. Wash the PBMC fraction with PBS.
8. Centrifuge at 350× *g*, 10 min, 21 °C with brakes.
9. Discard the supernatant and resuspend the PBMCs (visible as pellet) in PBS.
10. Repeat the PBS wash step.
11. Resuspend the PBMC pellet in an appropriate volume of PBS, aliquot in 1.5 mL reaction tubes and store them at −20 °C.

If PBMCs are to be cultured, all procedures must be carried out under sterile conditions in a cell culture hood.

#### 3.1.2. DNA Isolation from Blood Samples and Cell Cultures

DNA isolation was performed using the DNeasy Blood & Tissue kit (QIAGEN, Hilden, Germany) following the manufacturers’ instructions.

As test material, blood, PBMCs, primary cells or cell lines can be used. When using a frozen cell pellet, cells were allowed to thaw before PBS was added.

#### 3.1.3. DNA Isolation from Tissue Samples

Here, the DNeasy Blood & Tissue kit (QIAGEN, Hilden, Germany) was used, too. After 25 mg of tissue was cut into small pieces, the pieces were disrupted using a rotor–stator homogenizer for efficient DNA extraction.

#### 3.1.4. Nucleic Acid Extraction from Formalin-Fixed Paraffin-Embedded (FFPE) Tissue

The QIAamp DNA FFPE Tissue Kit (QIAGEN, Hilden, Germany) was used for the DNA extraction of FFPE tissue sections, while the RNeasy DSP FFPE Kit (QIAGEN, Hilden, Germany) was used for RNA extraction of these samples. All protocols were carried out according to the manufacturer’s recommendations.

#### 3.1.5. Nucleic Acid Extraction from Pig Skin

For DNA extraction, the DNeasy Blood and Tissue Kit (QIAGEN, Hilden, Germany) was used according to the manufacturer’s instructions. To extract the RNA of the pre-incubated samples, the RNeasy Lipid Tissue Mini Kit (QIAGEN, Hilden, Germany) was applied, and a DNase digestion was carried out using the RNase-Free DNase Set (QIAGEN, Hilden, Germany). The pig skin was cut into small pieces, treated with a mix of collagenase (final concentration 125 U/mL, Sigma-Aldrich, St. Louis, MO, USA) and hyaluronidase (final concentration 100 U/mL, Sigma-Aldrich, St. Louis, MO, USA), incubated for 30 min at 37 °C with shaking at 500 rpm, and finally centrifuged for 5 min at 1000× *g*. For more details, see [21].

#### 3.1.6. Nucleic Acid Quality Control and Quantification

DNA and RNA concentration and purity were measured by a NanoDrop spectrophotometer (Peqlab Biotechnologie GmbH).

1. Open the NanoDrop software (Nano Drop 1000, version 3.8.1) on the connected computer.
2. Initialize the instrument by loading 2 μL of nuclease-free water.
3. Perform a blank measurement using 2 μL of elution buffer.
4. Load 2 μL of the DNA sample and measure its concentration.
5. Record the DNA/RNA concentration as well as the 260/280 and 260/230 ratios to evaluate sample purity.

The absorbance ratio at 260 nm and 280 nm is commonly used to assess DNA purity. A ratio of approximately 1.8 is generally considered indicative of pure DNA and 2.0 for RNA. Lower values may suggest protein contamination. The 260/230 ratio is a secondary measure of nucleic acid purity. Ideal 260/230 values typically range from 2.0 to 2.2. Lower ratios may indicate the presence of contaminants such as EDTA, phenol or guanidine hydrochloride, which absorb strongly at 230 nm.

#### 3.1.7. RNA Isolation

For the isolation of RNA, the RNeasy Plus Mini kit (QIAGEN, Hilden, Germany) was used following the kit manufacturer’s instructions. A maximum of 1 × 10^7^ cells and less than 30 mg tissue can be used; the tissues have to be homogenized.

### 3.2. PCR Methods

#### 3.2.1. Primers and Probes Required

Primers and probes specific to the virus of interest were used to monitor their detection (Table 1). As a positive control, lab-based gene blocks were used as described in detail [22]. Four gene blocks were synthesized for specific virus genes. PERV-C was tested by conventional PCR, while all other viruses were tested by qPCR.

The following xenotransplantation-relevant viruses need to be tested in the donor animals: PCMV/PRV, PLHV-1, PLHV-2, PLHV-3, PCV1, PCV2, PCV3, PCV4, HEV, PERV-C, PPV, TTSuV1, TTSuV2, APPV and SARS-CoV-2. Testing for SARS-CoV-2 may not be necessary, as pigs are not susceptible to infection with this virus at present. However, this may change [35]. The recipient should be tested for any viruses known to be present in the donor pig. Even if the donor pig tests negative, additional testing of the recipient can help rule out the possibility that the virus was present in the donor at levels below the detection limit of the assay used. The recipient should be tested for PERV using the PERVpol PCR. To discriminate between true infection and the presence of PERV sequences in disseminated pig cells, screening for pig cells using the short interspersed nuclear element (SINE; PRE-1) PCR should be performed. The rationale of application and the enormous efficacy of this method were demonstrated when analyzing baboons transplanted with pig hearts [31].

#### 3.2.2. Conventional PCR

Conventional PCR was performed to determine the presence of PERV-C using a set of primers mentioned in Table 1. DreamTaq DNA polymerase (Thermo Fisher Scientific, Waltham, MA, USA) was used. All reactions were set up with a Biometra TRIO cycler (Analytik Jena, Jena, Germany).

Briefly, the sample was subjected to an initial denaturation for 10 min at 95 °C, followed by 45 amplification cycles of 95 °C for 15 s, annealing at 55 °C for 30 s, extension at 72 °C for 30 s and a final extension time at 72 °C for 5 min. As a positive control, liver tissue from a PERV-C-positive indigenous Greek black farm pig was used. Water served as a no-template control. A 1.5% agarose gel was used to detect the amplified DNA fragment of approximately 281 bp.

#### 3.2.3. Real-Time PCR

1. Perform the following steps in a clean master-mix-only room.
2. Clean the surface with DNase/RNase solution. Use filter tips all the time.
3. Prepare the primer–probe mix for the virus to be detected (Table A1).
4. Also prepare primer–probe mix for the internal control, i.e., GAPDH gene (Table A1).
5. Prepare a master mix in a new vial by adding SensiFAST probe no-ROX mix followed by virus primer–probe and GAPDH primer–probe, and top up the volume with nuclease-free water (Table A2).
6. Vortex the master mix vial and give it a short spin.
7. From now on, perform steps in a clean hood. Take a 96-well qPCR plate. Dispense 16 µL of master mix in each well, followed by 4 µL (100 ng) of DNA sample, making a total volume of 20 µL per well (prepare triplicates for each sample).
8. Also include a positive control, which is gene block DNA, and a no-template control (NTC), which is nuclease-free water.
9. Seal the plate carefully using the plastic sheet provided.
10. Give the plate a short spin in the centrifuge to spin down the liquid and to remove any air bubbles.
11. Place the plate in a qTOWER^3^ G qPCR cycler (Analytik Jena, Jena, Germany)
12. Set up the protocol for the respective virus (Table A3) and run it. At the end, observe the graph and calculate the Ct (threshold cycle) value.

### 3.3. Western Blot Analysis

#### 3.3.1. SDS-PAGE

Western blot analyses were performed to detect antiviral antibodies; their detection is especially important for herpesviruses such as PCMV/PRV, which may be in latency.

The Western blot analyses were performed using the corresponding recombinant viral proteins. The recombinant protein samples were dissolved in SDS-PAGE gel loading dye (Table A4), heated to 98 °C for 10 min, and centrifuged for 5 min at maximum speed.

The protein samples were run in SDS-PAGE gels with a suitable gel concentration, e.g., 17% for the glycoprotein B (gB) of PCMV/PRV and the p15E of PERV or 12% for the glycoprotein gp70 of PERV along with the protein marker. A Bio-Rad gel electrophoresis chamber (Bio-Rad, Hercules, CA, USA) was used with the following conditions: 80 V for 30 min, 120 V for 90 min. For blotting, the PVDF membrane (0.2 µm; Carl Roth, Karlsruhe, Germany) needed to be activated with absolute methanol for a few seconds and washed with transfer buffer (Table A4). Blotting was performed in a semi-dry electroblotter (Peqlab Biotechnologie GmbH, Erlangen, Germany) for 70 min at 40 mA. The membrane needed to be blocked with 5% milk powder-Tris-buffered saline with Tween20 (TBS-T) (Table A4) for 1 h at 4 °C with slow shaking. After blocking, the membrane was incubated with primary antibodies (i.e., serum or plasma samples prepared in blocking buffer, 1:50 up to 1:3000 dilution with TBS-T) overnight at 4 °C with slow shaking. Previously tested positive pig serum was used as a positive control (1:300 dilution); another pig serum that tested negative was used as a negative control. The membrane was washed 3 × 10 min with TBS-T and incubated with secondary antibody solutions prepared in blocking buffer for 1 h at room temperature with shaking. After this, the membrane was washed 3 × 10 min with TBS-T. The blot was developed using an ECL solution (i.e., 1:1) and a charge-coupled device (CCD) camera (Peqlab Biotechnologie GmbH).

#### 3.3.2. Western Blot Analysis PCMV/PRV

The recombinant R2 fragment of the PCMV/PRV glycoprotein (gB) was used to detect PCMV/PRV-specific antibodies as described [22,23]. The selection, cloning, production, purification and characterization of the recombinant proteins were described earlier [23]. Positive and negative control sera from infected and non-infected pigs were used. Goat anti-pig horseradish peroxidase (HRP) labeled antibodies (1:15,000 dilution) were used as secondary antibodies.

#### 3.3.3. Western Blot Analysis PLHV

Recombinant gB1 antigen of PLHV-1 was used to detect PLHV-1-specific antibodies as described [56]. Positive and negative control sera from infected and non-infected pigs were used. Polyclonal goat anti-pig immunoglobulin G (IgG)-HRP was used as a secondary antibody. Goat serum 3426, obtained by immunization with the purified gB1 antigen of PLHV, was used as a positive control serum.

#### 3.3.4. Western Blot Analysis PERV

Purified recombinant PERV proteins p15E and gp70 were used as antigens. The selection, cloning, production, purification and characterization of the recombinant proteins as well as the preparation of the positive control sera were described earlier [57]. For SDS-PAGE, 12% and 17% gels were prepared for gp70 and p15E, respectively. Pig and transplanted baboon sera were analyzed. Positive and negative control sera from goats immunized with the corresponding recombinant p15E and gp70 were used. As secondary antibodies, donkey anti-goat IgG HRP (1:20,000; MilliporeSigma, Bedford, MA, USA) and peroxidase AffiniPure goat anti-human IgG (H + L) (1:10,000; Jackson ImmunoResearch, West Grove, PA, USA), respectively, were used.

#### 3.3.5. Western Blot Analysis HEV

A recombinant genotype 3 (GT3) ORF2 HEV antigen (aa326–608, GT3-Ctr, 32 kDa) [18] containing the immunodominant region and a recombinant 44.5 kDa protein with a glutathione-S transferase (GST) tag fused to the ORF2 fragment (aa 452–617) (Prospec, Ness Ziona, Israel) as well as goat anti-pig IgG or goat anti-human IgG alkaline phosphatase-conjugated antibodies were used. Sera from an HEV-infected pig and an HEV-infected patient were used as positive controls, and sera from non-infected pigs were used as a negative control [18].

## 4. Expected Results

### 4.1. Results of PCR Analysis

Samples are classified as either positive or negative for a virus based on the Ct (cycle threshold) value. Regardless of the presence or absence of the target virus, the internal control gene (GAPDH) should always be detected. If GAPDH is not amplified, this indicates a failure in the assay—most likely due to the absence of DNA or poor sample quality.

The Ct value refers to the number of PCR cycles required for the fluorescent signal to exceed the background threshold, indicating detectable levels of DNA or RNA. A lower Ct value (e.g., 20) means that the target nucleic acid is abundant in the sample and requires fewer cycles to reach the detection threshold. Conversely, a higher Ct value (e.g., 30) indicates a lower initial amount of DNA or RNA, as more amplification cycles would be needed for detection.

Using gene blocks with the corresponding virus sequence, the sensitivity of the real-time PCRs is determined and has to be considered when analyzing donor pigs or recipients (Table 2). The standard curves can also be used to express the results in virus copy number/100 ng of DNA.

**Figure 1:**
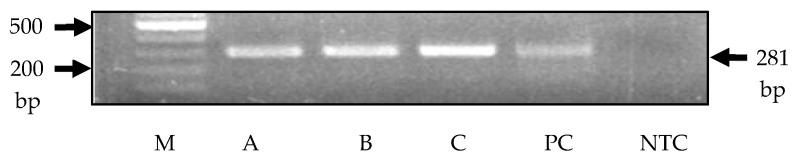
Detection of PERV-C in three piglets (A, B, C) using PCR analysis. M, marker Gene ruler 100 plus; PC, positive control, indigenous Greek black pig farm 1, pig 4; NTC, no-template control.

When we tested three pigs from a barrier facility, all animals were found negative using real-time PCRs for all tested viruses (Table 3). In addition, the animals were PERV-C-positive as detected using conventional PCR (Figure 1).

### 4.2. Results of Western Blot Analysis

#### 4.2.1. PCMV/PRV

Sera from pigs positive for PCMV/PRV will react with the recombinant protein and display a band at approximately 18 kDa. This corresponds to the band observed with the positive control serum. Since the intensity of the band reflects the antibody level, a semiquantitative statement is possible: stronger bands indicate higher antibody titers. In young piglets, a positive band may result from maternal antibodies acquired through colostrum, which wane over time when the piglet is not infected. To distinguish passive immunity from active infection, piglets should be tested at regular intervals. A positive result in an adult pig indicates an active or latent PCMV/PRV infection, and such animals should be excluded from use in xenotransplantation. Post-transplantation monitoring of the recipients should include testing of serum samples for signs of PCMV/PRV reactivation or new infection of the recipient.

**Figure 2:**
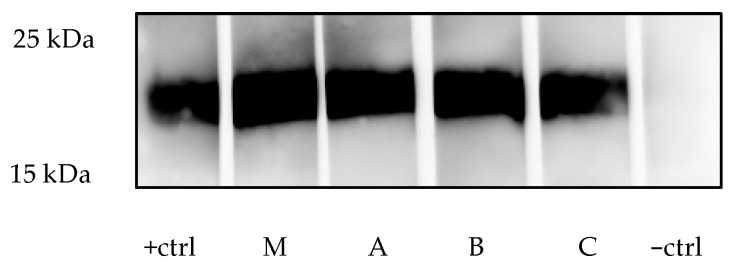
Western blot analysis of a mother pig (M) and three piglets (A, B, C). The piglets were 16 days old. Serum or plasma was used at 1:300 dilution; +ctrl, positive control, a positive pig; −ctrl, negative control, negative pig.

When we tested the three piglets for antibodies against PCMV/PRV, all three showed a strong reaction (Figure 2). In line with the age of the pigs, these antibodies were certainly obtained from the mother pig, which was also found to be positive (Figure 2). If the antibody titer decreases with time, the piglet is not infected.

#### 4.2.2. PLHV

If a pig is positive for PLHV antibodies, its serum will react with the recombinant glycoprotein B and show a band at approximately 35 kDa, consistent with the band observed in the positive control serum.

#### 4.2.3. PERV

Pigs do not produce antibodies against PERV; they are tolerant to these endogenous sequences. To make sure that the Western blot assay is functioning when screening recipients of pig organs, control sera have to be used; these control sera are obtained by immunization of goats with recombinant p15E or gp70 of PERV, which recognize the corresponding recombinant protein and show bands at approximately 12 kDa or 54 kDa, respectively [57].

#### 4.2.4. HEV

If a pig or a recipient is positive for HEV, the sera will react with one of the two related antigens representing parts of the capsid protein used as targets in Western blot analysis: first, a recombinant genotype 3 (GT3) ORF2 HEV antigen (aa 326–608, GT3-Ctr, 32 kDa) containing the immunodominant region, and second, a recombinant 44.5 kDa protein with a glutathione-S transferase (GST) tag fused to the ORF2 fragment (aa 452–617) [18].

## Figures and Tables

**Table 1:** Oligonucleotides for the primers and probes used in this study.

Virus	Primer/Probe	Sequence (5′ to 3′)	Reference
PCMV/PRV	PCMV-Fwd	ACTTCGTCGCAGCTCATCTGA	Müller et al., 2002 [43]
	PCMV-Rev	GTTCTGGGATTCCGAGGTTG	
	PCMV-Probe	6FAM-CAGGGCGGCGGTCGAGCTC-BHQ	
PLHV-1	PLHV-1 (1125)-Fwd	CTCACCTCCAAATACAGCGA	Chmielewicz et al., 2003 [44]
	PLHV-1 (1125) Rev	GCTTGAATCGTGTGTTCCATAG	
	PLHV-1 (1125) probe	6FAM-CTGGTCTACTGAATCGCCGCTAACAG-TAMRA	
PLHV-2	PLHV-2 (1155s)	GTCACCTGCAAATACACAGG	Chmielewicz et al., 2003 [44]
	PLHV-2 (1155as)	GGCTTGAATCGTATGTTCCATAT	
	PLHV-2 (1155) probe	6FAM-CTGGTCTACTGAAGCGCTGCCAATAG-TAMRA	
PLHV-3	PLHV-3 (210s)-Fwd	AACAGCGCCAGAAAAAAAGG	McMahon et al., 2006 [45]
	PLHV-3 (210as)-Rev	GGAAAGGTAGAAGGTGAACCATAAAA	
	PLHV-3 (210)-probe	6FAM-CCAAAGAGGAAAATC-MGB	
PCV1	PCV1 Fwd	AACCCCATAAGAGGTGGGTGTT	Chen et al., 2021 [46]
	PCV1 Rev	TTCTACCCTCTTCCAAACCTTCCT	
	PCV1 probe	6FAM-TCCGAGGAGGAGAAAAACAAAATACGGGA-BHQ1	
PCV2	PCV2 Fwd	CTGAGTCTTTTTTATCACTTCGTAATGGT	Chen et al., 2021, [46]
	PCV2 Rev	ACTGCGTTCGAAAACAGTATATACGA	
	PCV2 probe	ROX-TTAAGTGGGGGGTCTTTAAGATTAAATTCTCTGAATTGT-BHQ2	
PCV3	PCV3 Fwd	AGTGCTCCCCATTGAACG	Palinski et al., 2017 [47]
	PCV3 Rev	ACACAGCCGTTACTTCAC	
	PCV3 probe	6FAM-ACCCCATGGCTCAACACATATGACC-BHQ1	
PCV4	PCV4 Fwd	ATTATTAAACAGACTTTATTTGTGTCATCACTT	Chen et al., 2021 [46]
	PCV4 Rev	ACAGGGATAATGCGTAGTGATCACT	
	PCV4 probe	6FAM-ATACTACACTTGATCTTAGCCAAAAGGCTCGTTGA-BHQ1	
PPV-1	PPV-1 Fwd	CAGAATCAGCAACCTCACCA	Opriessnig et al., 2011 [48]
	PPV-1 Rev	GCTGCTGGTGTGTATGGAAG	
	PPV-1 probe	6FAM-TGCAAGCTT/ZEN/AATGGTCGCACTAGACA-BHQ1	
TTSuV1	TTSuV1-Fwd	CGAATGGCTGAGTTTATGCC	Xiao et al., 2012 [49]
	TTSuV1-Rev	GATAGGCCCCTTGACTCCG	
	TTSuV1-probe	6FAM-AACTGTCTA/ZEN/GCGACTGGGCGGGT-3IABkFQ	
TTSuV2	TTSuV2-Fwd	CGAATGGCTGAGTTTATGCC	Xiao et al., 2012 [49]
	TTSuV2-Rev	GATAGGCCCCTTGACTCCG	
	TTSuV2-probe	6FAM-AACAGAGCT/ZEN/GAGTGTCTAACCGCCTG-3IABkFQ	
HEV	HEV-Fwd	GGTGGTTTCTGGGGTGAC	Jothikumar et al., 2006 [50]
	HEV-Rev	AGGGGTTGGTTGGATGAA	
	HEV-probe	6FAM-TGATTCTCAGCCCTTCGC-BHQ	
APPV	APPV_5587-Fwd	CAGAGRAAAGGKCGAGTGGG	Postel et al., 2016 [34]
	APPV_5703-Rev	ACCATAYTCTTGGGCCTGSAG	
	APPV_CT-59 probe	6FAM-ACTACTATCCTTCGGGGGTAGTACCGA-BHQ1	
SARS-CoV-2	ORF1ab.F	GGCCAATTCTGCTGTCAAATTA	Dagotta et al., 2021 [51]
	ORF1ab.F	CAGTGCAAGCAGTTTGTGTAG	
	probe	6FAM-ACAGATGTCTTGTGCTGCCGGTA-BHQ1	
pGAPDH	pGAPDH-Fwd	GATCGAGTTGGGGCTGTGACT	Duvigneau et al., 2005 [52]
	pGAPDH-Rev	ACATGGCCTCCAAGGAGTAAGA	
	pGAPDH-probe	HEX-CCACCAACCCCAGCAAGAG-BHQ	
hGAPDH	hGAPDH-Fwd	GGCGATGCTGGCGCTGAGTAC	Behrendt et al., 2009 [53]
	hGAPDH-Rev	TGGTTCACACCCATGACGA	
	hGAPDH-probe	HEX-CTTCACCACCATGGAGAAGGCTGGG-BHQ1	
PERVpol	PERV pol Fwd	CGACTG CCCCAAGGG TTC AA	Yang et al., 2015 [29]
	PERV pol Rev	TCTCTCCTG CAA ATC TGG GCC	
	PERV pol probe	6FAM-CACGTACTG GAG GAG GGTCACCTG -BHQ1	
PRE-1	PRE-1 Fwd	GACTAGGAACCATGAGGTTGCG	Walker et al., 2003 [54]
	PRE-1 Rev	AGCCTACACCACAGCCACAG	
	PRE-1 probe	FAM-TTTGATCCCTGGCCTTGCTCAGTGG-BHQ1	
PERV-C *	PERV-C Fwd PERV-C Rev	CTGACCTGGATTAGAACTGG ATGTTAGAGGATGGTCCTGG	Takeuchi et al., 1998 [55]

Fwd = forward primer, Rev = reverse primer. * For the detection of PERV-C, other reliable PCR assays using other primers and probes can also be used.

**Table 2:** Sensitivity of different PCR-based methods for detecting pig viruses.

Virus	Method	Sensitivity (Copy Number per 100 ng DNA)	Sensitivity R^2^	Reference
PCMV/PRV ^a^	conventional PCR	15 copies		Morozov et al., 2016 [21]
	nested PCR	5 copies		
	real-time PCR	2 copies		
	real-time PCR	10 copies	0.9964	Jhelum et al., 2024 [58]
HEV3	real-time RT-PCR	150–200 copies		Morozov et al., 2015 [18]
	real-time RT-PCR	10 copies	0.9962	Jhelum et al., 2024 [58]
PCV2	real-time PCR	1 copy	0.9935	Jhelum et al., 2024 [58]
PCV3	real-time PCR	10 copies	0.9906	Jhelum et al., 2024 [58]
PCV4	real-time PCR	100 copies	0.9906	Jhelum et al., 2024 [58]
PLHV-1	real-time PCR	1 copy	0.9964	Jhelum et al., 2024 [58]
PLHV-2	real-time PCR	1 copy	0.9953	Jhelum et al., 2024 [58]
PLHV-3	real-time PCR	1 copy	0.9983	Jhelum et al., 2024 [58]
PPV1	real-time PCR	10 copies	0.9961	Jhelum et al., 2024 [58]

^a^ Please note the sensitivity of the nested PCR, which is not described here but can be used in addition; the method is described in [21].

**Table 3:** Screening of three pigs for xenotransplantation-relevant viruses using real-time PCR.

**Animal**	**PCMV/PRV**		**PLHV-1**		**PLHV-2**		**PLHV-3**		**PCV1**		**PCV2**		**PCV3**	
	**Virus** **ct**	**GAPDH ct**	**Virus** **ct**	**GAPDH** **ct**	**Virus** **ct**	**GAPDH** **ct**	**Virus** **ct**	**GAPDH** **ct**	**Virus** **ct**	**GAPDH** **ct**	**Virus** **ct**	**GAPDH** **ct**	**Virus** **ct**	**GAPDH** **ct**
A	n.d.	21.66	n.d.	21.12	n.d.	21.35	n.d.	21.92	26.66	21.91	n.d.	21.30	n.d.	21.98
B	n.d.	21.62	n.d.	21.06	n.d.	21.81	n.d.	21.88	n.d.	21.54	n.d.	21.36	n.d.	21.63
C	n.d.	21.94	n.d.	21.78	n.d.	21.33	n.d.	21.19	n.d.	21.05	n.d.	21.55	n.d.	21.37
**Animal**	**PCV4**		**PPV-1**		**TTSuV1**		**TTSuV2**		**APPV**		**HEV**		**SARS-CoV-2**	
	**Virus** **ct**	**GAPDH ct**	**Virus** **ct**	**GAPDH** **ct**	**Virus** **ct**	**GAPDH** **ct**	**Virus** **ct**	**GAPDH** **ct**	**Virus** **ct**	**GAPDH** **ct**	**Virus** **ct**	**GAPDH** **ct**	**Virus** **ct**	**GAPDH** **ct**
A	n.d.	21.32	n.d.	21.62	n.t.	n.t.	n.t.	n.t.	n.d.	30.76	n.d.	30.02	n.t.	n.t.
B	n.d.	21.59	n.d.	21.41	n.t.	n.t.	n.t.	n.t.	n.d.	30.98	n.d.	30.50	n.t.	n.t.
C	n.d.	21.40	n.d.	21.04	n.t.	n.t.	n.t.	n.t.	n.d.	30.01	n.d.	30.46	n.t.	n.t.

n.d., not detected; n.t. not tested because pigs cannot be infected with SARS-CoV-2 [35].

## Data Availability

Data is contained within the article or in the cited references.

## Appendix A

In this Appendix, details of the PCR methods are presented. This includes the primer and probe mixes (Table A1), the composition of the reaction mixes (Table A2) and the PCR programs used to detect porcine viruses and SINE sequences (Table A3).

**Table A1:** Primer–probe mix used to detect porcine viruses and cellular genes.

Compound	Amount (µL)
FAM mix for viruses PCMV/PRV, PLHV-1, PLHV-2, PCV1, PCV2, PCV3, PCV4, PPV1, TTSuV1, TTSuV2, HEV	
Fwd	10
Rev	10
probe	2.5
H_2_O	77.5
total	100
FAM mix for viruses PLHV-3, APPV, PERVpol	
Fwd	10
Rev	10
probe	5
H_2_O	75
total	100
FAM mix for PRE-1	
Fwd	10
Rev	10
probe	2
H_2_O	78
total	100
HEX mix for hGAPDH, pGAPDH	
Fwd	2.5
Rev	2.5
probe	2.5
H_2_O	92.5
total	100

**Table A2:** Preparation of reaction mix.

Compound	µL
2× SensiFast Probe No-ROX Mix	10
FAM mix for virus	1.8
HEX mix for GAPDH	1.8
H_2_O	2.4
Total	16
DNA template	4
Total reaction mix	20

**Table A3:** PCR programs used to detect porcine viruses and SINE sequences.

Virus	Time	Temperature	Number of Cyles
**PCMV/PRV**			
Inactivation	2 min	50 °C	
Initial Denaturation	10 min	95 °C	
Denaturation	15 s	95 °C	45 cycles
Annealing/Extension	1 min	60 °C	
**PLHV-1, PLHV-2, PPV1, PCV3**			
Inactivation	5 min	95 °C	
Denaturation	15 s	95 °C	45 cycles
Annealing	1 min	56 °C	
Extension	30 s	72 °C	
**PLHV-3**			
Inactivation	10 min	90 °C	
Denaturation	30 s	90 °C	45 cycles
Annealing/Extension	30 s	59 °C	
**PCV-1,2,4**			
Initial Denaturation	30 s	95 °C	
Denaturation	5 s	95 °C	45 cycles
Annealing/Extension	1 min	60 °C	
**HEV**			
Reverse Transcription	30 min	50 °C	
Inactivation	15 min	95 °C	
Denaturation	10 s	95 °C	45 cycles
Annealing	20 s	55 °C	
Extension	15 s	72 °C	
**APPV**			
Reverse Transcription	30 min	50 °C	
Inactivation	15 min	95 °C	
Denaturation	30 s	95 °C	40 cycles
Annealing	30 s	56 °C	
Extension	30 s	72 °C	
**SARS-CoV-2**			
Reverse Transcription	10 min	55 °C	
Inactivation	3 min	95 °C	
Denaturation	15 s	95 °C	45 cycles
Annealing/Extension	30 s	58 °C	
**TTSuV1, TTSuV2**			
Activation	2 min	50 °C	
Denaturation	10 min	95 °C	
Annealing	15 s	60 °C	45 cycles
Extension	15 s	60 °C	
**PERV-C**			
Initial Denaturation	10 min	95 °C	
Denaturation	15 s	95 °C	45 cycles
Annealing	30 s	55 °C	
Extension	30 s	72 °C	
Final Extension	5 min	72 °C	
**PERVpol**			
Inactivation	5 min	95 °C	
Denaturation	15 s	95 °C	45 cycles
Annealing	30 s	62 °C	
Extension	30 s	72 °C	
**PRE-1**			
Inactivation	5 min	95 °C	
Denaturation	15 s	95 °C	45 cycles
Annealing	30 s	60 °C	
Extension	30 s	72 °C	

## Appendix B

This appendix shows details of the SDS PAGE, mainly the preparation of buffers and solutions (Table A4).

**Table A4:** Preparation of buffers and solutions.

Solution	Ingredients	Remarks
**6× SDS-PAGE Sample Buffer**		
0.375 g	Tris	pH 6.8
12%	SDS	
60%	Glycerol	
0.6 M	DTT	
0.06%	Bromophenol blue	
**10× SDS-PAGE Running Buffer (1 L)**		
30.2 g	Tris	
144 g	Glycine	
10 g	SDS	
**10× Transfer Buffer**		**1** **× Transfer Buffer**
72 g	Glycine	1× 10× Transfer buffer
15.1 g	Tris	20% Methanol
2.5 g	SDS	Top up with water
**10× Tris-buffered saline (TBS) (1 L)**		pH 7.5
24.2 g	Tris	
87.6 g	Sodium chloride	
**TBS-T**		
0.05% Tween 20 in 1× TBS		
**Blocking buffer**		
5% milk powder in TBS-T		
